# Modulation of Lonp1 Activity by Small Compounds

**DOI:** 10.3390/biom15040553

**Published:** 2025-04-09

**Authors:** Giada Zanini, Giulia Micheloni, Giorgia Sinigaglia, Valentina Selleri, Anna Vittoria Mattioli, Milena Nasi, Ciro Leonardo Pierri, Marcello Pinti

**Affiliations:** 1 Department of Life Sciences, University of Modena and Reggio Emilia, 41125 Modena, Italy; giada.zanini@unimore.it (G.Z.); giulia.micheloni@unimore.it (G.M.); giorgia.sinigaglia@unimore.it (G.S.); valentina.selleri@unimore.it (V.S.); 2 National Institute for Cardiovascular Research—INRC, 40126 Bologna, Italy; annavittoria.mattioli@unibo.it; 3 Department of Quality-of-Life Sciences, University of Bologna, 40126 Bologna, Italy; 4 Department of Surgical, Medical, Dental and Morphological Sciences, University of Modena and Reggio Emilia, 41125 Modena, Italy; milena.nasi@unimore.it; 5 Department of Pharmacy-Pharmaceutical Sciences, University of Bari “Aldo Moro”, 70125 Bari, Italy; ciro.pierri@uniba.it

**Keywords:** Lon protease, proteostasis, protein quality control, cancer therapy, CDDO, bardoxolone, proteasome inhibitors, bortezomib, artemisinin

## Abstract

The Lon protease homolog 1 (LONP1) is an ATP-dependent mitochondrial protease essential for maintaining proteostasis, bioenergetics, and cellular homeostasis. LONP1 plays a pivotal role in protein quality control, mitochondrial DNA maintenance, and oxidative phosphorylation system (OXPHOS) regulation, particularly under stress conditions. Dysregulation of LONP1 has been implicated in various pathologies, including cancer, metabolic disorders, and reproductive diseases, positioning it as a promising pharmacological target. This review examines compounds that modulate LONP1 activity, categorizing them into inhibitors and activators. Inhibitors such as CDDO and its derivatives selectively target LONP1, impairing mitochondrial proteolysis, inducing protein aggregation, and promoting apoptosis, particularly in cancer cells. Compounds like Obtusilactone A and proteasome inhibitors (e.g., MG262) demonstrate potent cytotoxicity, further expanding the therapeutic landscape. Conversely, LONP1 activators, including Artemisinin derivatives and 84-B10, restore mitochondrial function and protect against conditions such as polycystic ovary syndrome (PCOS) and acute kidney injury (AKI). Future research should focus on improving the specificity, bioavailability, and pharmacokinetics of these modulators. Advances in structural biology and drug discovery will enable the development of novel LONP1-targeted therapies, addressing diseases driven by mitochondrial dysfunction and proteostasis imbalance.

## 1. Introduction

The Lon protease homolog 1 (Lonp1 or Lonp) is an essential ATP-dependent serine protease localized primarily in the mitochondrial matrix of eukaryotic cells. Lonp1 is member of the Lon family of proteases, whose first member was identified in *Escherichia coli* in the late 1960s as a key ATP-dependent protease involved in protein quality control and the degradation of abnormal or misfolded proteins. Early studies demonstrated its essential role in bacterial stress responses and intracellular protein homeostasis. By the 1990s, researchers had characterized homologs of Lon protease in eukaryotic cells, including mitochondria and peroxisomes, highlighting its conserved function in maintaining organellar proteostasis. The mitochondrial protease Lonp1 was found to regulate key aspects of mitochondrial function, including the turnover of oxidized proteins and the degradation of regulatory factors such as mitochondrial transcription factor A (TFAM). Because of its multiple functions in the mitochondria, an impairment of Lonp1 activity or regulation has been observed in many pathophysiological conditions, including several types of cancer [1,2,3,4,5,6,7], neurodegenerative diseases [8], cardiovascular diseases [9,10,11,12], and aging [13,14]. For this reason, there has been a growing interest in targeting Lon protease as a therapeutic strategy for various diseases, including cancer, neurodegeneration, and metabolic disorders. In this review, we critically re-evaluate data concerning compounds able to modulate LONP1 activity, either by inhibiting or enhancing its functions.

## 2. General Features of LONP1

**Figure 1:**
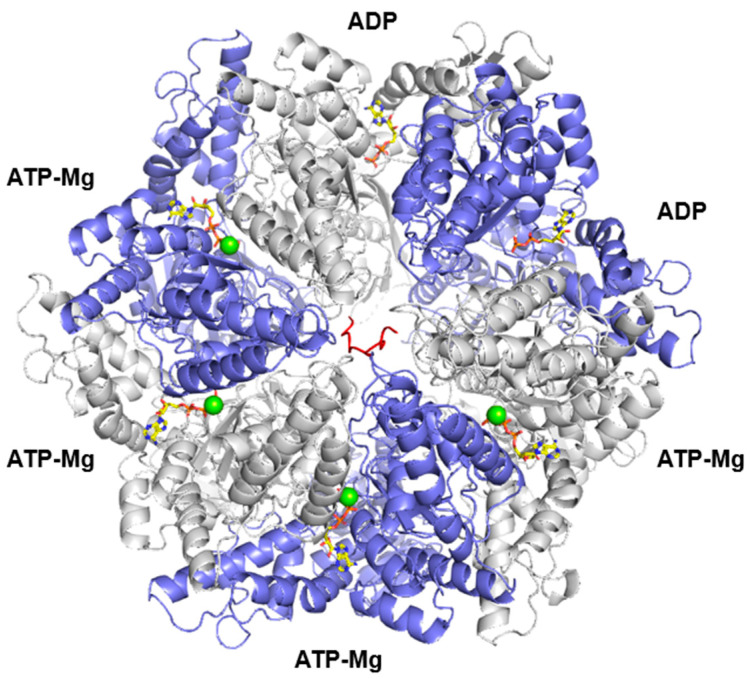
Three-dimensional structure of the hexameric LONP1 complex. Chains A, C, E are shown in white, whereas chains B, D, F are in violet; the enzyme is shown complexed with poly-alanine (red), according to 7kms.pdb. ATP and ADP in yellow sticks, whereas Mg are in green spheres.

Lonp1 is a multidomain protein composed of three main regions [15]. The substrate recognition domain, located at the N-term of the protein, ensures substrate specificity by recognizing and binding protein substrates through defined sequence motifs or structural features. The central, ATPase domain belongs to the AAA+ (ATPases Associated with various cellular Activities) superfamily and is essential for energy-dependent protein unfolding and translocation. ATP hydrolysis powers the conformational changes required for substrate processing. The proteolytic domain, containing a conserved serine–lysine catalytic dyad, mediates the degradation of misfolded, damaged, or unassembled proteins into short peptides [16]. Lonp1 works generally as a homohexamer—in some species, such as *Saccharomyces cerevisiae*, the enzyme is a homoheptamer. As shown in Figure 1, where human LONP1 homohexamer is depicted, the ATPase domains form a spiral staircase that interacts with the substrate, guiding it toward the proteolytic chamber [15]. This configuration highlights the coordinated action between the ATPase and protease domains during substrate degradation.

LONP1 also displays a chaperon-like activity, as the absence of LONP1 causes the accumulation of insoluble protein in the mitochondrial matrix. The anti-aggregation activity of LONP1 is primarily linked to its ATPase function rather than its protease activity. Cells expressing a protease-deficient LONP1 mutant (S855A) did not show signs of protein aggregation, whereas those expressing an ATPase-deficient mutant (K529A) accumulated insoluble proteins [17].

LONP1 also interacts with mitochondrial DNA (mtDNA) [18,19,20] and mtDNA-associated proteins, such as TFAM [21], modulating their stability and function [22]. This interaction supports mitochondrial genome replication, transcription, and packaging into nucleoids [23]. Finally, under conditions of oxidative stress or mitochondrial dysfunction, Lonp1 expression is upregulated by signaling pathways, including the integrated stress response (ISR) [24]. This adaptive mechanism enhances mitochondrial resilience by accelerating the degradation of damaged proteins.

As these different activities and functions can suggest, LONP1 is indispensable for multiple mitochondrial processes, contributing to cellular health and survival through several mechanisms, including (but not limited to) protein quality control, mtDNA maintenance, regulation of bioenergetics, response to cell stress and adaptation to stress conditions [25,26,27]. A detailed description of the mechanisms that link LONP1 functions and cell homeostasis goes far beyond the purpose of this review. Nevertheless, it is worth mentioning that the degradation of misfolded or oxidatively damaged proteins within the mitochondrial matrix is critical for preventing the accumulation of dysfunctional proteins, which could otherwise impair mitochondrial function and promote apoptosis [28,29]. This proteolytic activity is crucial for maintaining the integrity of the electron transport chain (ETC) and other mitochondrial complexes, thus promoting efficient mitochondrial respiration [5,30]. Under conditions of oxidative stress or nutrient deprivation, LONP1 helps reprogram mitochondrial metabolism, allowing cells to adapt to changing environmental conditions, such as hypoxic microenvironments, as often observed in solid tumors [2,6,31,32]. In various cellular models, including neurons and muscle cells, LONP1 has been shown to protect against ischemic and oxidative damage by maintaining mitochondrial function and preventing apoptosis. In agreement with its crucial roles in protection from and adaptation to stress, LONP1 is also implicated in the mitochondrial unfolded protein response (UPRmt) [30]—a protective mechanism activated in response to mitochondrial stress [33]. Under conditions of oxidative stress or nutrient deprivation, LONP1 expression is upregulated, facilitating the degradation of oxidatively damaged proteins and thus preventing the accumulation of toxic aggregates [34]. This adaptive response is vital for cellular survival, as it helps to restore mitochondrial function and proteostasis, particularly in cancer cells that often experience fluctuating metabolic demands. By interacting with key components of the mitochondrial import machinery, including translocase of inner mitochondrial membrane 44 (TIMM44), mitochondrial heat shock protein 70 (mtHSP70), and translocase of outer mitochondrial membrane 40 (TOMM40), LONP1 plays a role at the stage of protein translocation into the mitochondrial matrix, so favoring a proper import and folding of nuclear-encoded, mitochondrial proteins [17,35].

In agreement with its essential functions in mitochondria, *Lonp1* KO causes embryonic lethality in mice [2,36], and mutations of *LONP1* cause a severe genetic disease named CODAS (Cerebral, Ocular, Dental, Auricular, and Skeletal) syndrome. This rare, autosomal recessive multisystem disorder is characterized by developmental anomalies affecting multiple organ systems [37,38]. Clinical manifestations include cerebral malformations, congenital cataracts, dental abnormalities, external ear malformations, and skeletal dysplasia. Besides CODAS, *LONP1* mutations have been also identified as causative of classical mitochondrial diseases [39] and congenital diaphragmatic hernia [40].

## 3. Literature Research Methods

The present review adhered to the PRISMA (Preferred Reporting Items for Systematic Reviews and Meta-Analyses) guidelines to ensure a rigorous and transparent bibliographic research process in identifying compounds that modulate LONP1 activity. A systematic literature search was conducted across major scientific databases, including PubMed, Scopus, and Web of Science, using predefined keywords such as “LONP1 modulators”, “LONP1 activators”, “LONP1 inhibitors”, “mitochondrial protease inhibitors”, “Lon protease modulators”, “CDDO”, and related terms. The search was limited to peer-reviewed articles published in English, with no restrictions on publication year to capture both foundational and recent advancements. Inclusion criteria focused on studies that provided mechanistic insights into LONP1 modulation, identified specific compounds targeting LONP1, and described their effects in cellular or in vivo models. Exclusion criteria included conference abstracts and studies lacking direct evidence of LONP1 modulation.

## 4. Inhibitors of LONP1

**Figure 2:**
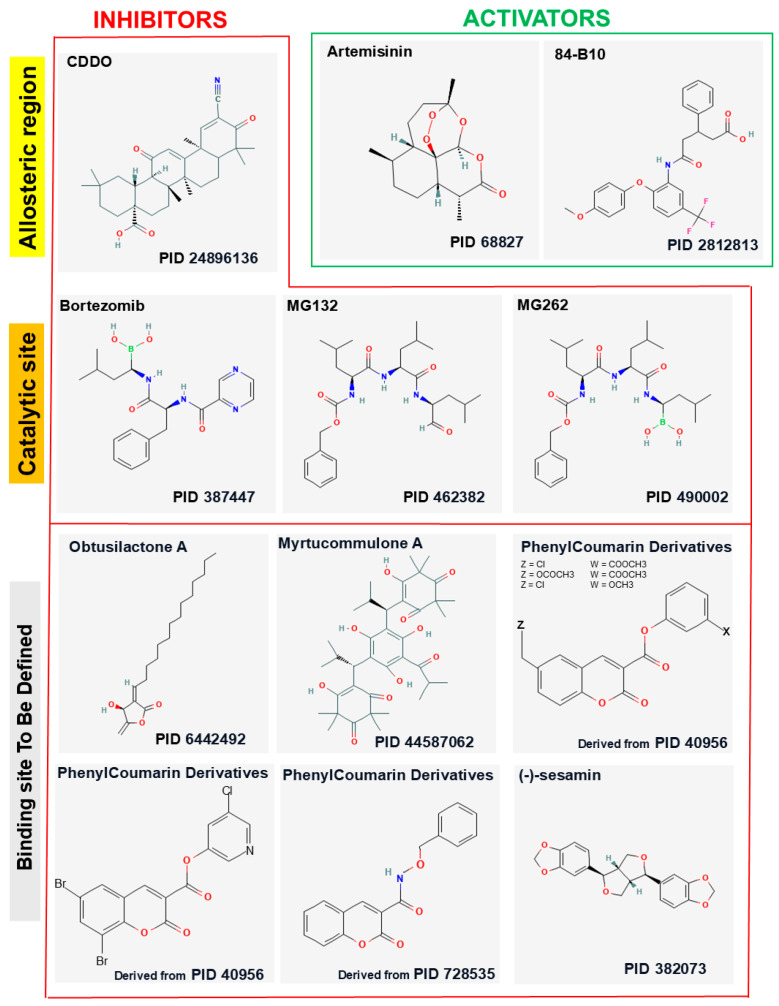
The 2D structures of 10 inhibitors and 2 activators were generated using the PubChem structure editor. The PubChem IDs are reported under the 2D structures of the indicated ligands. Phenylcoumarin analogs lacking on the PubChem database were derived starting from the indicated PIDs.

Several compounds, either isolated from plants or of synthetic origin, have been identified as potent inhibitors of LONP1; their structures are reported in the Figure 2. These inhibitors exert their biological effects primarily by interfering with mitochondrial proteostasis, leading to an accumulation of damaged proteins, mitochondrial dysfunction, and subsequent cell death.

### 4.1. CDDO and Its Derivatives

CDDO (2-Cyano-3,12-dioxooleana-1,9(11)-dien-28-oic acid) is a synthetic derivative of oleanolic acid—a pentacyclic triterpenoid isolated from medicinal plants belonging to various botanical families, including *Oleaceae* and *Lamiaceae*. Oleanolic acid is well known for its anti-inflammatory, antioxidant, and antitumor properties [41,42,43], but its limited potency has driven the development of more effective derivatives, including CDDO and its methylated or imidazolyl variants.

From a chemical perspective, CDDO features structural modifications that enhance its bioactivity: the introduction of a cyano (-CN) group at position C2 and the presence of reactive ketones at C3 and C12, which promote interactions with cellular nucleophiles [44,45]. These highly electrophilic groups are critical for CDDO biological activity, as they could facilitate covalent binding with specific target proteins, including mitochondrial enzymes and proteasomal components.

CDDO and its derivatives exhibit specific pro-apoptotic activity in B cells and cancer cells through the induction of protein aggregation within mitochondria [46,47]. Although it was previously shown that a biotinylated form of CDDO could form adduct with LONP1 [46], biochemical studies with purified LONP1 demonstrated that these molecules are non-competitive, reversible inhibitors of LONP1, which binds the proteins and blocks the ATPase activity of the enzyme [48]. Indeed, it has been shown that LONP1 is inhibited by a noncompetitive mechanism of inhibition by blocking ATP binding and hydrolysis and that CDDO derivatives inhibited not only the ATP-dependent protease activity but also the ATPase activity per se. Kinetics experiments showed that CDDO derivatives are slow binders of LONP1 and that the binding is reversible [48]. Structural studies have identified a hydrophobic pocket near the ATPase domain as the main binding site, with key residues (including F547, C576, and C637) playing crucial roles in compound binding [48]; a second binding site was identified in a more distal region from the ATP binding site. This effect is LONP1-specific, as CDDO did not inhibit the proteasome at concentrations able to block LONP1 ATPase activity. This inhibition causes the accumulation of undegraded proteins, which leads to the formation of electron-dense inclusion bodies within mitochondria, as observed via electron microscopy [47]. These protein aggregates induce mitochondrial stress, a loss of membrane potential, and the subsequent activation of the intrinsic apoptotic pathway [47]. Numerous studies have demonstrated that CDDO and its derivatives cause mitochondrial dysfunction through LONP1 inhibition [49]. Experiments on cancer cell lines treated with CDDO have shown several effects. First, CDDO determines the accumulation of damaged mitochondrial proteins, confirming the inhibition of LONP1-mediated protein turnover [47]. Damage to the mitochondrial respiratory chain results in reduced ATP production and increased reactive oxygen species (ROS) generation [47]. Inhibition of Lon protease by CDDO in *Caenorhabditis elegans* and humans has been also shown to improve mtDNA heteroplasmy, often observed during aging. Specifically, Yang and collaborators show that, in *Caenorhabditis elegans*, Lonp1 degrades stress-activated transcription factor-1 (ATFS-1) within functional mitochondria, which establishes an enriched interaction between ATFS-1 and ΔmtDNA, allowing the latter to be preferentially replicated. Inhibition of Lonp1 using CDDO was found to improve mtDNA replication over ΔmtDNA, reduce the heteroplasmy ratio and restore oxidative phosphorylation function in *Caenorhabditis elegans,* as well in human cybrid cells [23].

In addition to CDDO, two main derivatives, namely methylated CDDO (CDDO-Me, also known as bardoxolone-methyl) and imidazole CDDO (CDDO-Im), have been developed [48]. CDDO-Me features a methyl ester group at position C28, improving stability and cellular bioavailability. CDDO-Me is more potent than the parent compound in promoting apoptosis and inhibiting cell proliferation. CDDO-Im is characterized by the substitution of an imidazole group at position C28. This derivative increases the compound hydrophilicity, facilitating cellular uptake and enhancing efficacy in combination therapy contexts [48]. Despite these structural differences, both derivatives retain the ability to inhibit LONP1 and promote the accumulation of undegraded mitochondrial proteins. However, CDDO-Me and CDDO-Im demonstrate superior biological potency compared to the original CDDO, making them more promising forms for clinical applications [50].

It must be noted that CDDO-Me can also exert an effect on mitochondria independently from LONP1 by indirectly modulating the phosphorylation of dynamin-related protein 1 (DRP1), a protein crucial in the process of mitochondrial fission. The effects of CDDO-Me on mitochondrial dynamics and neuronal death has been studied in in the hippocampus, in a model of status epilepticus (SE) [51]: CDDO-Me increased the phosphorylation of DRP1 at Ser616 via the activation of extracellular-signal-regulated kinase 1/2 (ERK1/2) and c-Jun N-terminal kinase (JNK), which facilitated DRP1-mediated mitochondrial fission and selectively attenuated SE-induced CA1 neuronal death. This neuroprotectivity was independent of the inhibition of LONP1 mediated by CDDO-Me, as LONP1 knockdown led to widespread neuronal degeneration in the hippocampus without affecting mitochondrial dynamics [51].

CDDO-induced apoptosis is highly specific to cancer cells, particularly lymphomas and malignancies with high mitochondrial dependences [50]. A series of clinical trials evaluating the safety, efficacy, and pharmacokinetic (PK) properties of CDDO-Me have been conducted in healthy volunteers or patients with different diseases, with particular attention paid to chronic kidney disease (CKD), particularly those with type 2 diabetes mellitus (T2DM).

A Phase I trial conducted by Hong et al. investigated the pharmacokinetics of CDDO-Me in 47 patients with advanced solid tumors and lymphoid malignancies. The study aimed to determine dose-limiting toxicities, maximum tolerated dose, and optimal dosing for Phase II trials. Patients received oral doses of SDD CDDO-Me once daily for 21 days in a 28-day cycle, with pharmacokinetic assessments conducted at multiple time points [52]. The drug exhibited slow and saturable absorption, a long terminal half-life (39 ± 20 h at 900 mg), and dose-dependent nonlinearity at higher doses (600–1300 mg/day). Substantial interpatient variability was observed, with pharmacokinetic parameter variability ranging from 64 to 77% after the first dose and from 39 to 54% after the last dose. Despite this variability, once-daily dosing effectively maintained steady-state plasma concentrations within a controlled range [52]. The findings suggest that the inverse relationship between dose and absorption efficiency, along with the prolonged half-life, supports a once-daily dosing regimen for Phase II studies.

Although the CDDO-Me was developed to improve the low oral bioavailability of CDDO, the crystalline form of CDDO-Me still exhibited limited bioavailability, prompting the use of an amorphous spray-dried dispersion (SDD) formulation in clinical studies. It has been demonstrated that a 30 mg dose of SDD CDDO-Me provided higher bioavailability than a 150 mg dose of the crystalline form, while maintaining a comparable exposure profile in healthy individuals [53]. The Phase III BEACON (Bardoxolone Methyl Evaluation in Patients with Chronic Kidney Disease and Type 2 Diabetes Mellitus: The Occurrence of Renal Events) trial utilized this formulation to assess its therapeutic potential in CKD [54]. This was a Phase III, multicenter, randomized controlled trial involving 2185 patients with stage 4 CKD and T2DM. Despite CCDO-Me significantly improving estimated glomerular filtration rate (eGFR) and reducing the incidence of renal-related adverse events compared to placebo [54,55,56], the trial was prematurely terminated due to safety concerns, as a significant increase of hospitalization rates for heart failure and mortality was observed [57]. The AYAME study, another Phase III trial, aimed to evaluate the long-term effects of bardoxolone methyl on renal function in diabetic kidney disease patients. This study highlighted the drug ability to enhance eGFR significantly, but it also raised concerns about adverse cardiovascular events, echoing findings from the BEACON trial [58]

These trials (and several other preclinical and clinical studies not mentioned in this review due to limited space) highlighted that the use of CDDO and its derivatives presents several limitations [58,59,60,61,62]. At high concentrations, CDDO can induce cytotoxicity in normal cells as well, limiting its therapeutic window. Preclinical studies have revealed off-target effects associated with the formation of adducts with non-mitochondrial proteins. The original CDDO exhibits poor bioavailability and limited absorption into cancer cells due to its hydrophobic nature and low membrane permeability. Derivatives such as CDDO-Me and CDDO-Im, through structural modifications, show improved uptake but require further optimization [63,64]. Finally, rapid hepatic metabolism reduces the half-life of CDDO and its derivatives in vivo, necessitating strategies to enhance their stability and pharmacokinetic profile.

### 4.2. Obtusilactone

Obtusilactone A (OA) is a natural compound that falls under the class of butanolides or γ-lactones, characterized by a tetracyclic structure that includes a γ-lactone ring and an alkylidene moiety, as well as an aliphatic side chain. This compound is primarily isolated from plants within the *Lauraceae* family, belonging to *Cinnamomum* and *Lindera* genres. Notably, OA has been identified in the stems and leaves of *Cinnamomum kotoense*, *Cinnamomum tenuifolium*, and *Aiouea trinervis* [65,66,67]. The biosynthetic precursors of OA are believed to include structurally related butanolides, such as isoobtusilactone A and dihydroisoobtusilactone, which often co-occur in these plant species.

OA acts as a potent inhibitor of the LONP1 protease. Molecular docking studies revealed its interaction with the enzyme active site, where a carbonyl of the γ-lactone moiety of OA forms hydrogen bonds with the Ser855 and Lys898 residues [68]. This interaction is believed to inhibit LONP1 proteolytic activity, resulting in the accumulation of misfolded or damaged proteins within the mitochondria. Such disruption of mitochondrial protein homeostasis can trigger oxidative stress, apoptosis, and cell death, particularly in cancer cells that are highly dependent on LONP1 for survival. Specifically, OA-mediated inhibition of LONP1 has been demonstrated to induce caspase-3-mediated apoptosis in non-small-cell lung cancer (NSCLC) cell lines [68].

### 4.3. (-)-Sesamin

In the study that demonstrated the effect of OA on LONP1, it was also shown that (-)-sesamin has a similar effect. (-)-Sesamin is a bioactive lignan compound found primarily in sesame seeds and oil [69]. It has a chemical structure consisting of a furofuran ring system with two methylenedioxy groups [70,71]. The source and biosynthesis of (-)-sesamin have been extensively studied: sesame seeds are the main natural source of (-)-sesamin, which is produced via the phenylpropanoid pathway in the plant [70]. Genetic studies have identified key enzymes and genes involved in the biosynthesis of (-)-sesamin and related lignans in sesame. In particular, the cytochrome P450 enzyme CYP92B14 plays a critical role in the oxidation and conversion of (+)-sesamin to (+)-sesaminol and (+)-sesamolin [72]. Studies in rats showed that sesamin is absorbed in the liver 3–6 h after oral administration and redistributed to the serum and peripheral tissues after six hours from administration; it is detoxified by the liver via P450 and does not accumulate in the tissues [73]. In terms of the effects of (-)-sesamin, several studies have demonstrated its potent inhibitory activity against LONP1 [68]. (-)-Sesamin has been shown to directly interact with and inhibit LONP1 by binding to key residues in the active site, such as Ser855 and Lys898 [68]. This inhibition of LONP1 by (-)-sesamin leads to the induction of caspase-3-mediated apoptosis in various cancer cell lines, including NSCLC [68]. Beyond its effects on LONP1, (-)-sesamin has been reported to exhibit a wide range of other biological activities. It has demonstrated anti-inflammatory properties by suppressing the expression of inflammatory mediators such as interferon-β and inducible nitric oxide synthase [74]. (-)-Sesamin has also been shown to inhibit osteoclastogenesis and attenuate lipopolysaccharide-induced osteolysis through the suppression of ERK and NF-κB signaling pathways [75]. Additionally, (-)-sesamin and its metabolites have been found to possess antioxidant and vasorelaxant effects, which may contribute to its reported antihypertensive and cardioprotective properties [76,77,78].

The pharmacokinetics, pharmacodynamics, bioavailability, and metabolism of sesamin have been extensively investigated in both human and animal models. A study by Peñalvo et al. [79] assessed its bioavailability and absorption in humans, revealing high inter-individual variability in plasma lignan concentrations and identifying enterolactone as its major metabolite. Sesamin is absorbed and metabolized in the liver, though the exact mechanisms remain unclear. Pharmacokinetic parameters indicate rapid absorption (Cmax = 105 ± 11.7 nM at Tmax = 1 h) and a relatively short elimination half-life of approximately 2.08 h. Similar studies in Sprague–Dawley rats demonstrated that sesamin and its isomer episesamin exhibit peak serum concentrations at 7–9 h, with half-lives of 4.7 and 6.1 h, respectively.

As mentioned above, the metabolic pathways of sesamin involve cytochrome P450 enzymes (CYP2C9), leading to the formation of sesamin monocatechol (SC-1) and dicatechol (SC-2), which can further undergo methylation by catechol-O-methyltransferase (COMT) [80]. While SC-1 and SC-2 activate the Nrf/ARE pathway, their methylated variants do not, indicating a differential antioxidant potential [81]. Studies have also detected sesamin catechols in human urine, suggesting metabolism via glucuronidation by UDP-glucuronosyltransferase (UGT) enzymes [81]. Additionally, intestinal microflora convert sesamin into enterolactone and enterodiol, albeit at a slower rate than hepatic metabolism [82].

### 4.4. Myrtucommulone A

Myrtucommulone A (MC A) is a bioactive natural compound belonging to the class of acyl-phloroglucinols, first discovered and isolated from the Mediterranean plant *Myrtus communis* (common myrtle) [83]. *Myrtus communis* is an evergreen shrub widely distributed across the Mediterranean basin and North Africa, where it has been traditionally used in folk medicine for its anti-inflammatory, antimicrobial, and analgesic properties. Recent advancements in natural product chemistry enabled the isolation of MC A in pure form, revealing its rich pharmacological potential. Structurally, Myrtucommulone A is characterized by a polyphenolic core with two acyl side chains. It exists as three stereoisomeric forms, comprising two enantiomers and a meso form, which can interconvert under specific conditions [84]. This stereoisomeric versatility contributes to MC A dynamic chemical behavior and biological activity. Biosynthetically, MC A is thought to derive from simpler phloroglucinol precursors, such as nor-semimyrtucommulone (NSMC), through spontaneous non-enzymatic reactions. High-resolution chromatographic and spectroscopic techniques, including nuclear magnetic resonance and mass spectrometry, have been crucial in characterizing the structure and purity of MC A, achieving isolation at greater than 98% purity.

Myrtucommulone A has emerged as a potent modulator of mitochondrial proteostasis through its direct effects on mitochondrial chaperonins and proteases. Heat shock protein 60 (HSP60), a mitochondrial chaperonin essential for the folding and assembly of mitochondrial proteins, plays a pivotal role in LONP1 regulation, particularly under conditions of heat shock [85]. MC A selectively binds to HSP60, inhibiting its chaperone activity and impairing its ability to stabilize LONP1. The inhibition of HSP60 has significant downstream effects on LONP1, leading to its destabilization and subsequent aggregation. When LONP1 becomes dysfunctional due to MC A-induced HSP60 inhibition, its protective proteolytic activity is compromised, resulting in protein aggregation and mitochondrial stress [85].

Given its potential as inhibitor of mitochondrial functions, the distribution and pharmacokinetics of MC were investigated in cellular models and in rats, to assess its absorption, metabolism, and distribution. MC A demonstrated high permeability in Caco-2 cells, suggesting good oral bioavailability. Extensive phase I metabolism was observed, with over 50% of MC A metabolized in human liver microsomes within 15 min and only 10% remaining after 120 min. In rats, MC A exhibited a plasma level of 258.6 ng/mL after 1 h, with a t½ of 10.3 h following oral administration (4 mg/kg). Physiologically based pharmacokinetic modelling suggested that MC A accumulates in highly perfused tissues, including the liver, skin, and brain.

### 4.5. MG132

MG132 (carbobenzoxy-Leu-Leu-leucinal) is a potent and reversible peptide aldehyde proteasome inhibitor. Structurally, it consists of a tripeptide backbone—Z-Leu-Leu-Leu—where “Z” represents a carbobenzoxy protective group. MG132 was developed as an inhibitor of calpain activation in the framework of a series of studies aimed at clarifying the mechanism of erythrocyte membrane fusion in the presence of Ca^++^, and it was subsequently shown to primarily target the proteasome [86,87,88,89]. By inhibiting the chymotrypsin-like activity of the proteasome catalytic 20S core, MG132 prevents the degradation of polyubiquitinated proteins, leading to their accumulation within the cell. The aldehyde group at the C-terminus plays a critical role in its function by forming a reversible covalent bond with the active site of proteolytic enzymes.

Beyond its canonical role as proteasome inhibitor, MG132 has been shown to inhibit LONP1 activity in the mitochondria. In an effort to clarify the mechanisms underpinning the degradation of steroidogenic acute regulatory protein (StAR), it has been shown that mature StAR exhibits a turnover rate of approximately 4.2 h in the mitochondrial matrix—significantly faster than that of other mitochondrial proteins—and that MG132 (but not the specific proteasome inhibitor epoxomicin) blocks the degradation of mature StAR within mitochondria [90]. This observation suggested that MG132 could target mitochondrial proteases. Subsequently, in vitro assays using purified murine StAR and LONP1 confirmed that MG132 effectively inhibited the ATP-dependent Lon-mediated degradation of StAR and increased StAR half-life by at least threefold. Further in vitro characterization using FITC-casein as a substrate confirmed the inhibitory effects of MG132, with an IC50 of 20 μM [91]. The effect on LONP1 is protein-specific, as MG132 did not inhibit the mitochondrial protease CLPXP even at concentrations up to 400 μM [29]. The inhibitory effect likely does not depend on the substrate, as the effect of MG132 on LONP1 has been observed with other substrates, such as ornithine transcarbamylase (OTC) or HSP60 [92].

### 4.6. MG262

MG262 is a synthetic proteasome inhibitor that belongs to the class of peptidyl boronates. As stated above, peptide aldehydes such as MG132 were initially used as inhibitors of cellular proteases but were later shown to efficiently work as proteasome inhibitors. From these molecules, peptide boronates were developed [93,94], as the substitution of boronic acid for the aldehyde moiety produces more potent proteasome inhibitors. In this context, MG262 was originally developed as a synthetic analog of the natural product MG132 [95]. Like MG132, MG262 contains a peptide backbone with a boronic acid “warhead” that covalently binds to and inhibits the active site Ser of the proteasome catalytic subunits. The synthesis of MG262 involves coupling an amino acid recognition moiety with the boronic acid electrophilic group to create the peptidyl boronate structure [95]. MG262 is a highly potent and selective inhibitor of the proteasome, with a mechanism of action that differs from non-peptidic proteasome inhibitors [95,96,97]. In addition to its proteasome inhibitory effects, MG262 has been shown to have anti-inflammatory and anti-proliferative properties in various cell types and disease models [98]. The inhibition of proteasome activity by MG262 requires the binding of ATP, in contrast to other peptidyl boronates that can inhibit serine/threonine proteases in an ATP-independent manner [97]. This ATP-dependent mechanism of inhibition is thought to be a distinctive feature of MG262 and other peptidyl boronates. Indeed, MG262 has been shown to inhibit the bacterial form of Lon protease from *Salmonella enterica* serovar Typhimurium in an ATP-dependent manner [95,96,97]. After discovering its inhibitory function in bacteria, MG262 has been used in different studies as a human LONP1 inhibitor, either on the purified enzyme or in cell lines [99], even if no deep characterization of MG262-LONP1 interaction has been provided.

### 4.7. Bortezomib

Bortezomib is a dipeptide boronate originally developed as a proteasome inhibitor, similarly to MG262. Bortezomib is widely used in the treatment of multiple myeloma and other malignancies. The elimination of the internal amino acid residue of tripeptide boronic acid inhibitors improves the specificity of the molecules, since dipeptides are inefficiently bound by serine proteases. Similarly to MG132 and MG262, bortezomib interacts with and inhibits LONP1 [49].

Lee et al. demonstrated that bortezomib inhibits LONP1 with an IC50 of 17 nM, which is comparable to its inhibition of the 20S proteasome (IC50 of 2.3 nM) [48]. This study utilized X-ray crystallography and cryo-electron microscopy to show that bortezomib binds to the active site of LONP1, indicating a direct interaction that could influence the protease activity. Further investigations explored the structure–activity relationship of bortezomib in relation to LONP1 specificity, confirming that bortezomib has an affinity for LONP1 that is comparable to that for proteasome, despite the structural and sequence differences within the LONP1 and chymotrypsin pockets [100]. Interestingly, in the same study, a series of bortezomib analogues were developed in order to increase specificity for LONP1, and one of those compounds (((*R*)-1-((*R*)-2-(2,4-Dimethyloxazole-5-carboxamido)pentanamido)-4-phenylbutyl)boronic acid) showed higher affinity for LONP1 and very low affinity for the proteasome. Further insights into the mechanism of inhibition by bortezomib have been obtained by the study of the effect of this drug (and MG262) on LonC—a bacterial form of Lon-like protease that degrades protein substrates in an ATP-independent manner [101,102]. In contrast with observations in previous other studies, at least in the case of MG262 [95,96,97], it was shown that both inhibitors bind covalently to Ser582, a residue crucial for the catalytic activity of Lon protease, confirming that they block the enzyme activity by forming covalent adducts with the proteolytic site, independently from ATPase domain. Bortezomib forms a tetrahedral adduct with Ser582 (homologue of LONP1 Ser855), with the boronate hydroxyl groups and hydrogen bonded to Lys625 and to Ser582 [103]. Thus, it is likely that bortezomib and MG262 can inhibit LONP1 via two independent mechanisms; it would be interesting to determine which is the most important at the concentrations found to be active on LONP1 by Lee et al. [48], and if the two mechanisms impact the chaperone and proteolytic roles of LONP1 within mitochondria differently. Additionally, the work of Maneix et al. emphasizes the role of LONP1 in mediating resistance to proteasome inhibitors like bortezomib in multiple myeloma cells [104]. They found that the inhibition of LONP1 could sensitize cancer cells to bortezomib, suggesting that targeting LONP1 may enhance the efficacy of proteasome inhibitors in cancer therapy. This dual targeting approach could be particularly beneficial in overcoming resistance mechanisms that often limit the effectiveness of bortezomib. However, it must be underlined that the inhibition of LONP1 by bortezomib can also lead to increased levels of mitochondrial substrates, such as cytochrome P450 family 11 subfamily A member 1 (CYP11A1), which are otherwise degraded by LONP1 [105]. This accumulation may have downstream effects on cellular metabolism and apoptosis, further complicating the therapeutic landscape of bortezomib treatment.

Because of its effects on proteasome, bortezomib has been extensively studied in clinical trials for the treatment of different types of cancer and is nowadays used in the treatment of multiple myeloma and mantle cell lymphoma; whether LONP1 inhibition contributes to the therapeutic benefit of bortezomib or is an off-target effect is still a matter of debate. Bortezomib exhibits complex pharmacokinetic properties that influence its therapeutic efficacy and safety profile. Bortezomib is typically administered intravenously or subcutaneously, with bioavailability dependent on the route of administration. Intravenous administration results in rapid systemic distribution, with a peak plasma concentration of approximately 100 ng/mL within 5 min, whereas subcutaneous administration results in a lower C_max of 20–30 ng/mL, achieved over 30–60 min, potentially improving tolerability [106]. Bortezomib undergoes extensive hepatic metabolism, primarily via cytochrome P450 enzymes, particularly CYP3A4, with minor contributions from CYP2C19 and CYP1A2. Metabolic biotransformation leads to inactive metabolites, which are primarily excreted via the hepatobiliary route, while renal clearance plays a minimal role in drug elimination [107]. The drug follows a biphasic elimination pattern, with an initial rapid decline in plasma concentration with a half-life of **10–30 min** followed by a prolonged terminal half-life of 5–15 h. The pharmacokinetic profile of bortezomib also underpins its dose-dependent toxicities, particularly peripheral neuropathy, which is more pronounced with intravenous administration due to higher peak plasma concentrations. The lowest toxic dose (TDLo) in mice was 5 mg/kg/14D following the intraperitoneal administration of an intermittent dose and 1.6 mg/kg/12D following the subcutaneous administration of a continuous dose.

### 4.8. Coumarinic Derivatives

As MG132, MG262, and bortezomib are inhibitors more effective of proteasome than LONP1, Bayot and colleagues tried to identify LONP1 inhibitors that had no crossed effect on proteasomes [108]. They successfully identified four coumarinic derivatives (Figure 2) that inhibited LONP1 in a rapid, time-dependent, reversible fashion at very low concentrations. The kinetics of inhibition and reactivation of the enzyme are compatible with a formation of an acyl enzyme, which leads to a transient inactivation. All these compounds did not show inhibitory activity of the yeast proteasome. The authors proposed that these molecules could represent specific and efficient inhibitors of LONP1 [108]. However, no further studies regarding LONP1 reported the use of these compounds.

## 5. Activators of LONP1

Compounds that enhance LONP1 activity have been explored as therapeutic agents to mitigate diseases associated with mitochondrial dysfunction. Although fewer activators than inhibitors of LONP1 have been identified, some molecules have proven to be potentially interesting for pharmacological modulation of LONP1 functions.

### 5.1. Artemisinin and Its Derivatives

Artemisinin is a sesquiterpene lactone with a distinctive endoperoxide bridge (C-O-O-C) that underpins its biological activity (Figure 2). It was first isolated in 1972, during efforts to address growing resistance of *Plasmodium falciparum* to chloroquine, from *Artemisia annua*, a medicinal plant traditionally used in China to treat febrile illnesses [109]. The chemical structure of artemisinin served as the foundation for developing first-generation derivatives, including dihydroartemisinin (DHA), obtained through the conversion of carbonyl to hydroxyl groups [109].

Beyond their anti-malarial applications, artemisinin derivatives have shown promise in addressing hyperandrogenemia associated with polycystic ovary syndrome (PCOS) [105]. In addition, artemisinin appears to modulate the acetyl-transferase activity of specific variants of the mitochondrial carnitine O-acetyl transferase (CRAT), associated with a case of early-onset Leigh syndrome, either on recombinant proteins or on patient cell lysates [110]. In murine models of PCOS, the artemisinin derivative artemether demonstrated significant improvements in hallmark features of the syndrome, including the reduction of hyperandrogenism, the normalization of irregular estrous cycles, the restoration of polycystic ovarian morphology, and improved fertility outcomes.

In an effort to identify the mechanisms underlying the therapeutic effects of artemisinins, it was found out that they can suppress ovarian androgen synthesis by targeting CYP11A1, the key enzyme catalyzing the first step of androgen biosynthesis [105]. Accordingly, artemisinin treatment led to a marked reduction in CYP11A1 levels, effectively curbing androgen production. Further mechanistic investigations revealed that this effect is LONP1-mediated: artemisinin enhanced the interaction between LONP1 and CYP11A1, promoting the proteolytic degradation of CYP11A1 [105]. Interestingly, the androgen-inducing factor human chorionic gonadotropin (hCG) was found to disrupt the LONP1-CYP11A1 interaction, leading to increased CYP11A1 levels and androgen synthesis in PCOS. A docking model of LONP1 with artemisinin proposed that artemisinin can bind residues of the catalytic pocket at the level of residues D852, S855, M810, P854, K898, and G893 [10].

Importantly, it was shown that the decrotonylation of LONP1 at Lys390 or its downregulation were associated with mitochondrial dysfunction in PCOS, and that lower levels of LONP1 were found in blood samples from patients with PCOS [111]. The study by Liu and collaborators confirmed these observations: LONP1 was significantly downregulated in PCOS models, resulting in elevated CYP11A1 levels and enhanced androgen biosynthesis [105]. This finding suggests that the reduced activity of LONP1 contributes to the hyperandrogenic-state characteristic of PCOS. Supporting this, an overexpression of LONP1 in ovarian tissue effectively suppressed androgen production, further validating LONP1 as a critical regulator of ovarian androgen synthesis. The translational potential of artemisinin was further supported by a pilot clinical trial in 19 PCOS patients. Treatment with DHA led to significant improvements in clinical outcomes, including reduced hyperandrogenism, decreased anti-Müllerian hormone levels, improved ovarian morphology, and the normalization of menstrual cycles [109]. An issue raised by this approach in clinical settings is the short half-life of artemisinin (1–2 h) after oral administration [112]. Indeed, patients involved in the pilot study by Liu et al. took dihydroartemisinin 40 mg thrice daily, suggesting that more durable artemisinin derivatives or different administration routes (e.g., transdermal) should be considered for the treatment of a chronic disease such as PCOS [105].

### 5.2. 84-B10

A second molecule identified as an activator of LONP1 is 84-B10 ((chemical name: 5-[[2-(4-methoxyphenoxy)-5-(trifluoromethyl) phenyl] amino]-5-oxo-3-phenylpentanoic acid), a synthetic 3-phenylglutaric acid derivative. This molecule was firstly characterized as a compound able to alleviate cisplatin-induced nephrotoxicity, by restoring mitochondria homeostasis and inhibiting mtROS-induced ferroptosis [113].

Its function as an LONP1 activator has been discovered in the framework of a study exploring the complex interconnection between mitochondrial impairment and mitochondrial 3-hydroxy-3-methylglutaryl-CoA synthase (HMGCS2) in the pathogenesis of CKD [114]. After showing that HMGCS2 is a possible substrate of LONP1, the authors explored the therapeutic potential of 84-B10 as a LONP1 activator, targeting its role in renal fibrosis and tubular epithelial cell protection. Through molecular docking and protease activity assays, 84-B10 emerged as the most effective LONP1 activator among 19 candidates, exhibiting strong binding affinity (KD = 312.5 nM) and promoting the degradation of LONP1 substrate TFAM. Structural analysis revealed key interactions between 84-B10 and LONP1, including hydrogen bonds and a salt bridge [114].

This role of 84-B10 as an activator of LONP1 has been further confirmed in another study, where it was tested against aristolochic acid nephropathy (AAN), a form of renal toxicity linked to *Aristolochiaceae*-derived aristolochic acids (AAs). Using RNA-seq analysis, researchers identified mitochondria and peroxisomes as key cellular components affected by 84-B10 treatment in AAN models. The compound preserved mitochondrial structure, improved mitochondrial respiration, enhanced fatty acid β-oxidation via increased expression of critical transporters and enzymes, and reduced mtROS. Similarly, 84-B10 restored peroxisomal fatty acid β-oxidation and redox balance. As knocking down LONP1 attenuated the protective effects of 84-B10, the effects of this compound were likely due to a LONP1-dependent mechanism [115].

Structural modeling suggests that 84-B10 binds to the catalytic domain of LONP1, enhancing its peptidase activity. In particular, 3D molecular modelling analysis showed that 84-B10 can form four conventional H-bonds with TRP770, ASP852, LYS898, and GLY893, as well as one salt bridge with LYS898. Surface plasmon resonance studies have confirmed the physical interaction between 84-B10 and LONP1 [114].

Given its promising therapeutic potential for treating AKI and CKD, a physiologically based pharmacokinetic (PBPK) model has been recently set up to predict drug distribution and therapeutic outcomes of 84-B10 [116]. The study employs ultra-high-performance liquid chromatography-tandem mass spectrometry (UHPLC-MS/MS) for quantification, validating the specificity, linearity, precision, and stability of the method. Pharmacokinetic analysis in rats indicates rapid absorption and elimination, with widespread tissue distribution, particularly in the intestine, stomach, liver, kidney, and lungs. A whole-body PBPK model was constructed and validated and further applied to simulate 84-B10 pharmacokinetic profile in healthy and CKD patients. The results indicated a slower elimination rate in CKD patients due to reduced renal blood flow and glomerular filtration rate, leading to higher plasma drug exposure and lower kidney concentrations compared to healthy individuals. Under repeated dosing conditions, steady-state concentrations were achieved within a day, with notable differences in peak kidney concentrations between CKD patients and healthy individuals.

The main features of the inhibitors and activators of LONP1 are summarized in Table 1.

## 6. Conclusions and Future Perspectives

LONP1 is a vital regulator of mitochondrial function, proteostasis, and cellular homeostasis, and its dysregulation has been implicated in numerous diseases, including cancer, metabolic disorders, and reproductive syndromes. Pharmacological modulators of LONP1—comprising inhibitors and activators—demonstrate significant therapeutic potential, yet several challenges must be addressed before their clinical application becomes available. LONP1 inhibitors have been associated with enhanced apoptosis due to increased reactive oxygen species (ROS) production, leading to anti-tumorigenic effects by limiting cell proliferation and motility, which is particularly relevant in resistant cancer types [5]. Additionally, inhibitors such as CDDO-Me have demonstrated synergistic potential with proteasome inhibitors like carfilzomib in multiple myeloma, enhancing protein stress and therapeutic efficacy [104]. Since LONP1 overexpression contributes to tumor aggressiveness in colorectal and lung cancers, its inhibition may mitigate malignancy by disrupting survival mechanisms associated with this protease [1,2]. First, their cross-reactivity with the proteasome, as observed with compounds such as MG132, MG262, and bortezomib, despite the structural differences in their catalytic sites, poses serious challenges in terms of toxicity; this off-target activity underscores the urgent need for selective inhibitors that minimize unintended effects. Second, pharmacokinetic challenges such as drug bioavailability and mitochondrial accumulation remain unresolved, as evidenced by the development of CDDO-Me from CDDO, which has yielded suboptimal results. Third, LONP1 inhibition raises concerns for potential non-specific toxicity due to its role in essential cellular processes, leading to mitochondrial dysfunction, metabolic dysregulation, and increased susceptibility to cellular stress. Fourth, resistance mechanisms may also emerge, as cancer cells could develop compensatory pathways that enable survival under prolonged LONP1 inhibition [104]. Moreover, responses to LONP1 inhibition vary among different cancer types, and in some cases, the suppression of LONP1 may even enhance resilience rather than induce cell death [4]. Given these risks, the therapeutic use of LONP1 inhibitors is likely more effective when combined with other compounds that mitigate mitochondrial toxicity. For instance, in multiple myeloma, LONP1 inhibition has been shown to enhance the efficacy of proteasome inhibitors, reducing drug resistance and improving overall therapeutic outcomes [104]. Similarly, in gliomas, combining LONP1 inhibitors with chemotherapeutic agents has led to increased sensitivity to treatment while minimizing off-target effects [117].

On the other hand, LONP1 activators could offer therapeutic benefits in non-oncological contexts, such as in reproductive and metabolic disorders. Activators like artemisinin and 84-B10 have been shown to provide protective effects in PCOS and AKI, respectively, highlighting their potential applications beyond cancer therapy. One particularly compelling case for LONP1 activation lies in the treatment of CODAS syndrome, a rare autosomal recessive genetic disorder caused by pathogenic variants in LONP1, which results in a partial or total impairment of its functionality. Although the precise mechanisms by which LONP1 mutations disrupt mitochondrial function and lead to the broad spectrum of clinical manifestations remain under investigation, studies using LONP1 knockout cellular models indicate that mitochondrial dysfunction and impaired protein homeostasis contribute to disease pathology. Enhancing LONP1 residual activity through small-molecule activators such as 84-B10 could partially restore LONP1 function and help recover mitochondrial homeostasis in CODAS patients. More broadly, LONP1 activators promote protein homeostasis by enhancing the degradation of misfolded proteins—a mechanism crucial for maintaining mitochondrial function and preventing disease progression [118]. Activation of LONP1 has also been linked to neuroprotective effects in neurodegenerative diseases, where it facilitates the clearance of toxic proteins, improving neuronal survival [119]. Additionally, the chaperone-like functions of LONP1 can assist in proper protein folding, potentially aiding cellular recovery under stressful conditions such as hypoxia or nutrient deprivation [15]. Nevertheless, LONP1 activation is not without risks, as increased LONP1 activity could inadvertently promote cancer cell survival in aggressive tumor types by enhancing resilience under stress conditions, thereby reducing the effectiveness of conventional treatments [1]. Furthermore, increased LONP1 activity could contribute to oncogenic processes by allowing genetically predisposed cells to evade apoptosis, raising concerns about therapy-induced tumor progression [5]. The therapeutic window for LONP1 activation is also limited, as excessive activation may disrupt physiological balance, compromising the viability of surrounding healthy tissues and restricting its clinical applicability [120].

Overall, the modulation of LONP1 presents both opportunities and challenges in various disease contexts. Inhibitors show promise in tumor suppression but pose risks of toxicity, resistance, and off-target effects, while activators may support cellular health, particularly in CODAS syndrome and neurodegenerative disorders, but could inadvertently contribute to tumor progression. Achieving an optimal therapeutic strategy requires a nuanced approach, with careful consideration of disease type and stage, the specific role of LONP1 in different pathologies, and the development of highly selective and bioavailable pharmacological agents. Advances in structural biology and drug design will be instrumental in overcoming these hurdles, paving the way for innovative therapies targeting mitochondrial dysfunction and proteostasis.

## Figures and Tables

**Table 1:** List of compounds known to modulate LONP1 activity.

Compound	Origin/Source	Mechanism(s) of Action	References
CDDO	Synthetic derivative of oleanolic acid	Binds LONP1 next to ATPase active inhibits its proteolytic function	[47,48,49]
CDDO-Me	Synthetic (methyl ester of CDDO)	Binds LONP1 next to ATPase active inhibits its proteolytic function. More potent than CDDO	[48,50,51]
CDDO-Im	Synthetic (Imidazolide derivative of CDDO)	Binds LONP1 next to ATPase active inhibits its proteolytic function. More potent than CDDO	[48,50]
Obtusilactone A	Extracted from *Cinnamomum kotoense*, *Cinnamomum reticulatum*, and *Aiouea trinervis*	Directly interacts with and inhibits LONP1	[68]
(-)-Sesamin	Extracted from sesame (*Sesamum indicum*) seeds	Directly interacts with and inhibits LONP1	[68]
Myrtucommulone A	Extracted from *Myrtus communis* (common myrtle)	Inhibits HSP60, destabilizing LONP1 under thermal stress, leading to LONP1 aggregation	[85]
MG132	Synthetic proteasome inhibitor	Inhibits indirectly LONP1 proteolytic activity when ATP is bound by LONP1	[91,92]
MG262	Synthetic proteasome inhibitor	Inhibits LONP1 proteolytic activity, when ATP is bound by LONP1	[95,96,97]
Bortezomib	Synthetic compound	Directly binds to the active site of LONP1 and inhibits its protease activity	[49,100,105]
Coumarinic derivatives	Synthetic derivatives of coumarinic acid	Bind to the active site of LONP1 and form a stable acyl-enzyme	[108]
Artemisinin	Extracted from *Artemisia annua*	Enhances the interaction between LONP1 and its substrates, such as CYP11A1	[10,105]
84-B10	Synthetic compound	Directly binds to the catalytic domain of LONP1, enhancing its peptidase activity	[114,115]

## Data Availability

Not applicable .
